# Effects of age-dependent changes in cell size on endothelial cell proliferation and senescence through YAP1

**DOI:** 10.18632/aging.102236

**Published:** 2019-09-05

**Authors:** Tadanori Mammoto, Yu-Suke Torisawa, Megan Muyleart, Kathryn Hendee, Charles Anugwom, David Gutterman, Akiko Mammoto

**Affiliations:** 1 Department of Pediatrics, Medical College of Wisconsin, Milwaukee, WI 53226, USA; 2 Cardiovascular Center, Medical College of Wisconsin, Milwaukee, WI 53226, USA; 3 Department of Medicine, Medical College of Wisconsin, Milwaukee, WI 53226, USA; 4 Department of Cell Biology, Neurobiology and Anatomy, Medical College of Wisconsin, Milwaukee, WI 53226, USA; 5 Hakubi Center for Advanced Research, Kyoto University, Kyoto 615-8540, Japan; 6 Department of Micro Engineering, Kyoto University, Kyoto 615-8540, Japan

**Keywords:** aging, cell size, cell proliferation, senescence, angiogenesis

## Abstract

Angiogenesis – the growth of new blood capillaries- is impaired in aging animals. Biophysical factors such as changes in cell size control endothelial cell (EC) proliferation and differentiation. However, the effects of aging on EC size and the mechanism by which changes in cell size control age-dependent decline in EC proliferation are largely unknown. Here, we have demonstrated that aged ECs are larger than young ECs and that age-dependent increases in EC size control EC proliferation and senescence through CDC42-Yes-associated protein (YAP1) signaling. Reduction of aged EC size by culturing on single-cell sized fibronectin-coated smaller islands decreases CDC42 activity, stimulates YAP1 nuclear translocation and attenuates EC senescence. Stimulation of YAP1 or inhibition of CDC42 activity in aged ECs also restores blood vessel formation. Age-dependent changes in EC size and/or CDC42 and YAP1 activity may be the key control point of age-related decline in angiogenesis.

## INTRODUCTION

Angiogenesis plays important roles in organ development, regeneration and pathology [1, 2]. Angiogenic signaling (e.g., vascular endothelial growth factor (VEGF), Tie2, FGF, HIF1α) and endothelial cell (EC) proliferation are attenuated in aging animals and age-dependent decline in angiogenesis leads to the development of aging-associated diseases, including cardiovascular diseases, Alzheimer’s disease, osteoporosis, diabetes, and COPD [3–5]. Although most studies of angiogenesis have focused on soluble angiogenic factors and signaling molecules, biophysical factors such as cell size and geometry, cell-cell and cell-matrix interactions, extracellular matrix (ECM) stiffness, and blood flow also play important roles in angiogenesis [6, 7]. We have reported that EC shape and size control EC proliferation [8]. Culturing ECs on substrates of different stiffness or at the different densities also changes cell size and shape, and consequently modulates angiogenic gene expression and regulates EC proliferation, behaviors, and function [9–11]. ECM stiffness [12] and blood flow [5], which change cell size and shape, are altered in aged tissues with epithelial cells and fibroblasts being generally larger than those in younger tissues [13–16]. Senescent cells [14] or highly passaged cells [13], which mimic the phenotype of aged cells, are also larger than non-senescent or lower passaged cells. However, the direct effects of EC size on age-dependent changes in EC proliferation and suppression of angiogenesis have not been explored.

A Hippo signaling transducer, Yes-associated protein (YAP1) acts as a transcriptional co-activator and controls organ size and regeneration (*e.g.,* liver, heart, intestine, muscle, lung) [17, 18]. YAP1 stimulates angiogenesis and vascular function through various signaling pathways, including angiopoietin2 (Ang2), microfibrillar-associated protein 5, matrix metalloproteinase 2 (MMP2), VE-cadherin, and peroxisome proliferator-activated receptor gamma, coactivator 1 alpha (PGC1α) [19–23]. YAP1 is a mechanosensitive gene, and EC size and geometry control YAP1 activity [18, 24, 25]. Other mechanical forces such as rigidity and topology of the ECM [24, 26, 27] and shear stress [18, 21, 28, 29] that consequently alter cell shape and size also control the activity of YAP1. However, the physiological relevance of the direct effects of changes in EC size on YAP1 activity and the underlying mechanism remain unclear. Knockdown of YAP1 induces cellular senescence [30] and suppresses angiogenesis and organ regeneration (e.g., liver) in aged adults [31]. Deregulation of YAP1 signaling also contributes to aging-associated diseases such as COPD [32], pulmonary fibrosis [18, 33], and Alzheimer’s disease [34, 35]. Rho-GTPase CDC42 senses mechanical forces, induces filopodia formation and regulates cellular adhesions and polarity in various types of cells including ECs and fibroblasts [36, 37]. CDC42 controls angiogenesis by changing multiple morphogenetic processes of EC sprouting [38, 39]. It has been known that CDC42 activity is higher in aged tissues [40–42] and that CDC42 controls YAP1 activity, and vice versa during retinal vascular development [20, 22] and lung epithelial regeneration [43].

Here we have demonstrated that aged ECs are larger than young ECs. Older ECs exhibit higher CDC42 activity and lower YAP1 activity compared to younger ECs. Reduction of aged EC size using the microcontact printing system decreases CDC42 activity, stimulates YAP1 nuclear translocation, inhibits EC senescence, and reverses EC proliferation. Modulation of CDC42 and YAP1 activity restores angiogenesis in aged tissue and could be a promising therapeutic strategy for aging-associated diseases.

## RESULTS

### Aged mouse and human ECs are larger than young ECs

**Figure 1:**
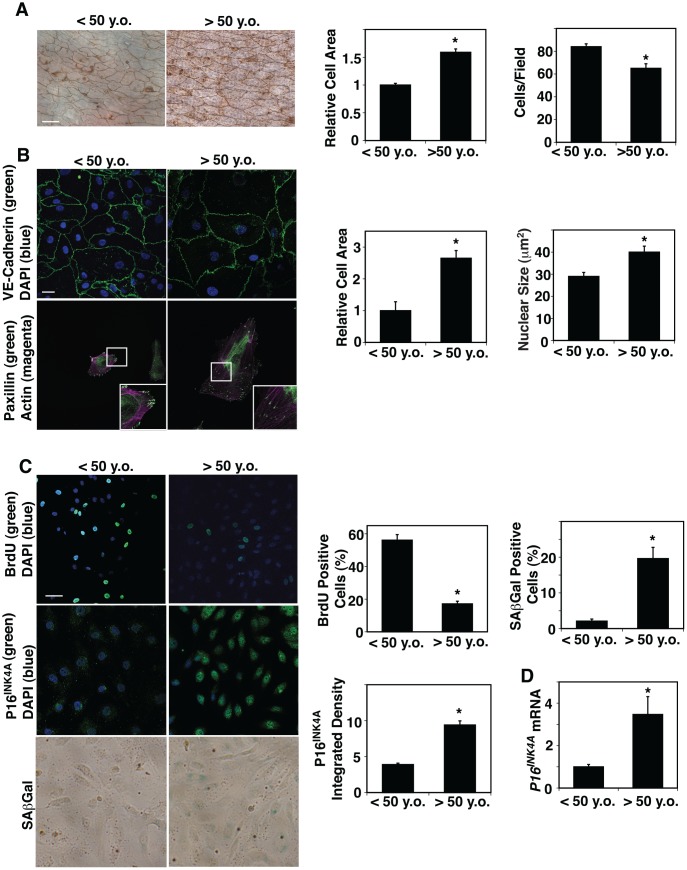
**Age-dependent changes in human adipose EC size, proliferation and senescence.** (**A**) Silver nitrate-stained <50 years old (<50 y.o.) and >50 years old (>50 y.o.) human adipose tissue blood vessels. Scale bar, 20 μm. Graphs showing quantification of cell area (*left*) and cell density (*right*) in blood vessels dissecting from <50 y.o. and >50 y.o. human adipose tissues (n=27, 28, mean ± s.e.m., *, p<0.05). (**B**) Immunofluorescence (IF) micrographs showing VE-cadherin-positive cell-cell junctions and DAPI (*top*) and paxillin-positive focal adhesions and actin stress fiber formation (*bottom*). Scale bar, 20 μm. Graphs showing quantification of cell area (*left*) and nuclear size (*right*) of ECs isolated from <50 y.o. and >50 y.o. human adipose tissues (n=5, mean ± s.e.m., *, p<0.05). (**C**) IF micrographs showing BrdU^+^ ECs isolated from <50 y.o. and >50 y.o. human adipose tissues (*top*). IF micrographs showing P16^INK4A^-positive ECs isolated from <50 y.o. and >50 y.o. human adipose tissues (*middle*). Micrographs showing SAβGal-stained ECs isolated from <50 y.o. and >50 y.o. human adipose tissues (*bottom*). Scale bar, 20 μm. Graphs showing quantification of BrdU^+^, P16^INK4A+^, and SAβGal-stained ECs isolated from <50 y.o. and >50 y.o. human adipose tissues (n=5, mean ± s.e.m., *, p<0.05). (**D**) Graph showing *P16^INK4A^* mRNA levels in ECs isolated from <50 y.o. and >50 y.o. human adipose tissues (n=7, mean ± s.e.m., *, p<0.05).

ECM stiffness [12] and blood flow [5], which are altered in aged tissues, change EC size and shape. However, the direct effects of aging on EC size in blood vessels have not been explored. We dissected small blood vessels with a length of circumference of 300 μm (a diameter of approximately 50 μm) from human adipose tissues of various ages (Table 1) and measured EC size in blood vessels by staining with silver nitrate [44, 45], which stains cell-cell junctions, ex vivo. The areas of ECs of small blood vessels in adipose tissues of age older than 50 years old (>50 y.o.) were 1.6-times larger than those from younger adults (< 50 y.o.) (Figure 1A). In contrast, EC density was 25% lower in the aged adipose tissue blood vessels (Figure 1A). Isolated aged human adipose ECs cultured on fibronectin (FN)-coated tissue culture dishes were also larger (2.6-fold) compared to young ECs when analyzed using VE-cadherin staining (Figure 1B). The size of nuclei was also 1.4-times larger in cultured aged human adipose ECs compared to young ECs (Figure 1B). There was no significant difference in the actin stress fiber structures (e.g. thickness, numbers) in young vs. old ECs (Figure 1B), however, a major focal adhesion protein, paxillin, which was specifically localized in the punctate form at the distal ends of actin stress fibers in young adipose ECs, was distributed along the actin fibers in the cytoplasm in aged ECs (Figure 1B). Consistent with others’ reports [46, 47], EC proliferation measured by BrdU nuclear incorporation was lower by 69% in ECs isolated from aged human adipose tissue, while cellular senescence detected by P16^INK4A^ immunocytochemical (ICC) analysis and SA-β galactosidase (Gal) staining increased in aged human adipose ECs; the intensity of P16^INK4A^ and SA- βGal-positive cells increased by 2.2- and 10.7-times in aged vs. younger human ECs (Figure 1C). The mRNA levels of *P16^INK4A^* detected by qRT-PCR were also 3.5-times higher in aged human ECs (Figure 1D).

**Table 1:** Sample demographics.

**Sample demographics (n=55)**	**Young (< 50 y.o., n=27)**	**Old (> 50 y.o., n=28)**
Gender, Male/Female	11 (40%)/16 (60%)	13 (46%)/15 (54%)
Age, year (mean ± s.e.m)	38.55±1.26	62.07±1.77
Body mass index (mean ± s.e.m)	33.31±2.00	30.00±1.32
Underlying diseases		
Coronary artery disease	2 (7%)	7 (25%)
Hypertension	4 (15%)	14 (50%)
Hyperlipidemia	1 (4%)	10 (36%)
Diabetes mellitus	3 (11%)	7 (25%)
Atrial fibrillation	0	4 (14%)
Myocardial infarction	0	0
None of the above	6 (22%)	6 (21%)

It is known that microenvironment surrounding ECs and gene expression patterns are different among capillaries, large blood vessels and pulmonary circulation. Therefore, we examined the size of the ECs from different origins ex vivo: large blood vessels from mouse descending aorta and pulmonary blood vessels from mouse pulmonary artery (PA). When we dissected mouse PA (diameter 100 μm) from 2 months (2M) vs. 24M old mouse lungs and measured EC size using silver nitrate staining [44, 45], 24M old mouse PA ECs were 2.3-times larger than those in 2M old mice ex vivo (Supplementary Figure 1A), while EC density was 29% lower in the aged PA (Supplementary Figure 1A). Similar trends were observed in descending aorta; 24M old mouse aortic EC size was 1.6-times larger, while EC density was 29% lower compared to those in 2M old mouse aortic ECs (Supplementary Figure 1A). Isolated 24M old mouse lung ECs, which include capillary ECs, cultured on FN-coated tissue culture dishes were also 1.4-times larger compared to 2M old mouse lung ECs (Supplementary Figure 1B). Paxillin was also distributed more along the actin fibers in the cytoplasm in 24M old mouse lung ECs (Supplementary Figure 1B). Consistent with human adipose tissue-derived ECs (Figure 1), EC proliferation measured by BrdU nuclear incorporation was inhibited by 54%, while cellular senescence detected by P16^INK4A^ ICC analysis and SA-β Gal staining was 2.6- and 3.8-times higher, respectively, in 24M old mouse lung ECs (Supplementary Figure 1C). The mRNA and protein levels of P16^INK4A^ also increased by 2.2- and 3.9- times, respectively, in aged mouse lung ECs (Supplementary Figure 1E, 1F). These results suggest that aging increases EC size, alters cytoskeleton structures, and induces cellular senescence in both mouse and human ECs.

### CDC42-YAP1 signaling mediates the effects of aged cell size on EC proliferation and senescence

**Figure 2:**
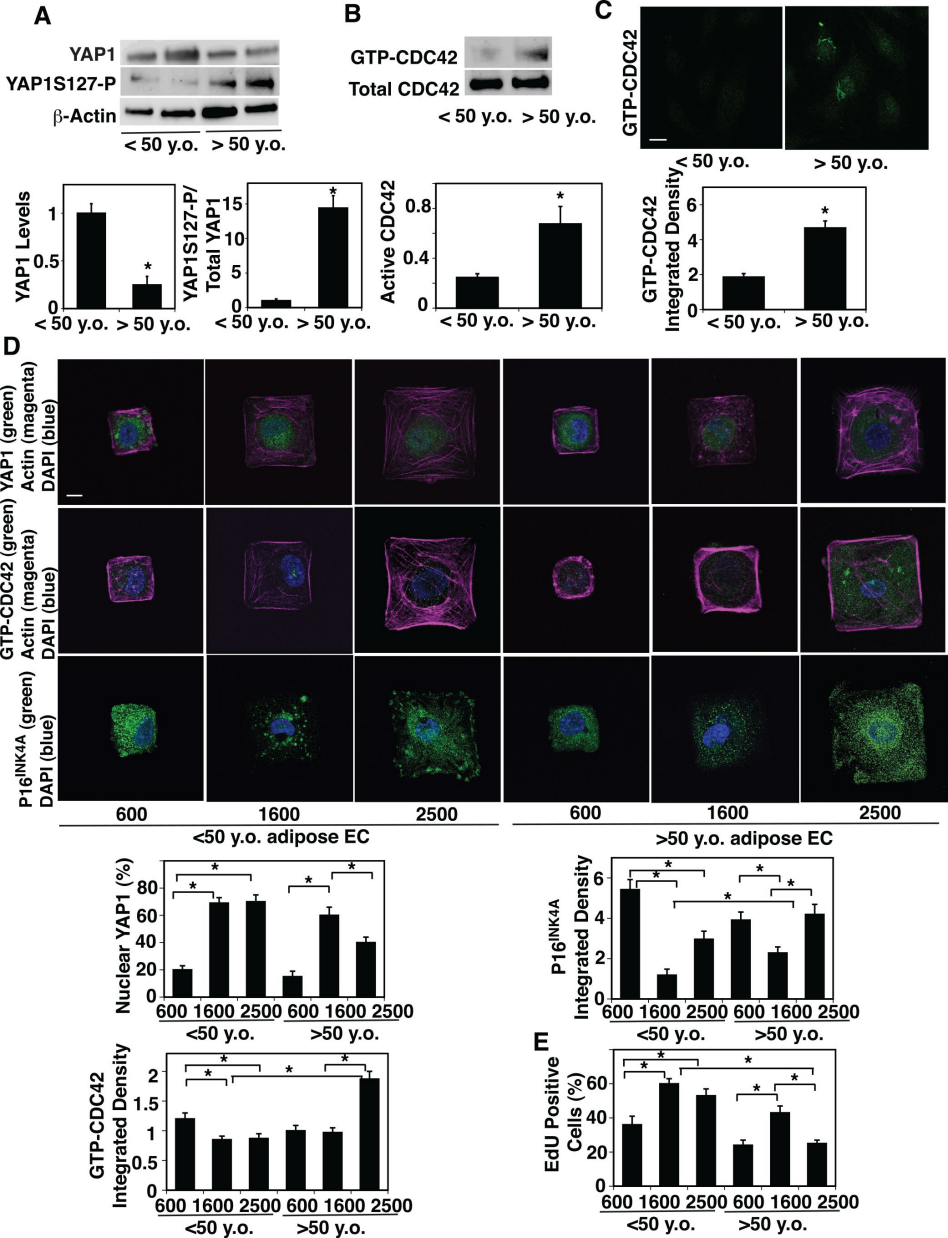
**Age-dependent changes in YAP1 and CDC42 activity in human adipose ECs.** (**A**) Representative immunoblots showing YAP1, YAP1S127 phosphorylation and β-actin protein levels in ECs isolated from <50 y.o. and >50 y.o. human adipose tissues (*top*). Graphs showing the quantification of immunoblots (*bottom,* n=6, mean ± s.e.m., *, p<0.05). (**B**) Representative immunoblots showing GTP-CDC42 and total CDC42 protein levels in ECs isolated from <50 y.o. and >50 y.o. human adipose tissues (*top*). Graph showing the quantification of immunoblots (*bottom,* n=6, mean ± s.e.m., *, p<0.05). (**C**) IF micrographs showing the levels of GTP-CDC42 in ECs isolated from <50 y.o. or >50 y.o. human adipose tissues (*top*). Graph showing quantification of the GTP-CDC42 levels in ECs isolated from <50 y.o. and >50 y.o. human adipose tissues (n=5, mean ± s.e.m., *, p<0.05). (**D**) IF micrographs showing YAP1 nuclear localization (green), actin (magenta), and DAPI (blue, *top*), GTP-CDC42 levels (green), actin (magenta) and DAPI (blue, *middle*), and P16^INK4A^ (green) and DAPI (blue, *bottom*) in ECs isolated from <50 y.o. or >50 y.o. human adipose tissues cultured on FN-coated island of different sizes. Scale bar, 10 μm. Graphs showing quantification of nuclear YAP1 (*left top*), GTP-CDC42 integrated density (*left bottom*) , and P16^INK4A^ integrated density (*right top*) (n=7, mean ± s.e.m., *, p<0.05). (**E**) Graph showing quantification of EdU-positive cells (n=7, mean ± s.e.m., *, p<0.05).

It has been known that YAP1 senses cell size and controls cell proliferation [18, 24, 26, 27, 29]. Given that EC size increases during aging (Figure 1, Supplementary Figure 1), we next examined whether YAP1 mediates the effects of age-dependent increases in cell size on EC proliferation and senescence. The mRNA and protein levels of YAP1 were 81% and 79% lower, respectively, in aged human adipose ECs and 78% and 91% lower, respectively, in mouse lung ECs compared to young ECs (Figure 2A, Supplementary Figure 2A, 2B). Phosphorylation of YAP1 at the serine127 (S127) residue by large tumor suppressor (LATS) sequesters YAP1 to the cytoplasm and has a potent role in suppressing YAP1 activity and subsequently decreases gene expression that controls cell proliferation [29, 48]. YAP1S127 phosphorylation was 14.4-times higher, and YAP1 was excluded from the nucleus and inactive in old human adipose ECs compared to young ECs (Figure 2A, Supplementary Figure 2C).

CDC42 controls YAP1 activity and regulates alveolar epithelial stem cell proliferation in stretching cells in vitro and in the mouse lung after pneumonectomy (PNX) in vivo, in which mechanical forces are dramatically altered [43]. It has been reported that CDC42 activity is higher in aged tissues [40–42]. Therefore, we next examined the effects of aging on CDC42 activity in ECs. Consistent with others’ reports [40–42], CDC42 activity measured by PAK pull-down assay increased by 3.1-times in aged human adipose ECs compared to younger human adipose ECs (Figure 2B). ICC analysis confirmed the results; intensity of GTP-CDC42 was 2.3-times higher in aged human adipose ECs compared to young human adipose ECs (Figure 2C).

To directly analyze whether EC size controls YAP1 activity, we prepared microcontact-printed substrates consisting of square FN-coated islands (600–2500 μm^2^) surrounded by non-adhesive regions, in which we directly stamped FN (50 μg/ml) onto activated polydimethylsiloxane-coated cover slips and blocked unstamped areas with Pluronic F-127 [49, 50]. When we cultured young human adipose ECs on FN-coated printed islands of different sizes [49, 50], YAP1 was localized in the nucleus (active form) on the islands of medium size (1600 μm^2^) or larger islands (2500 μm^2^), while YAP1 was in the cytosol and inactive when cultured on the smaller islands (600 μm^2^) (Figure 2D). In contrast, when we cultured aged human adipose ECs on the large islands (2500 μm^2^), YAP1 was excluded from the nucleus, while YAP1 was in the nucleus on the medium size islands (1600 μm^2^) (Figure 2D), suggesting that EC size controls YAP1 nuclear localization in a distinct way depending on the EC age. Similar trends were observed in 2M vs. 24M old mouse lung ECs (Supplementary Figure 2D, 2E); when we cultured 2M old mouse lung ECs on FN-coated printed islands of different sizes [49, 50], YAP1 was localized in the nucleus and active on the medium and large islands (900 vs., 2500 μm^2^), while YAP1 was in the cytosol and inactive when cultured on the smaller islands (600 μm^2^) (Supplementary Figure 2D, 2E). In contrast, when we cultured 24M old mouse lung ECs on the large islands, YAP1 was excluded from the nucleus, while YAP1 was in the nucleus on the medium size islands (Supplementary Figure 2D, 2E).

CDC42 senses various mechanical forces and its activity was higher in aged human adipose tissue-derived ECs (Figure 2B, 2C). Therefore, we also examined whether age-dependent changes in EC size control CDC42 activity. ICC analysis revealed that the levels of GTP-CDC42 were higher in aged ECs cultured on the large island (2500 μm^2^) compared to those in young ECs cultured on the medium-large island (1600-2500 μm^2^, Figure 2D). Reduction of aged EC size by culturing on the medium size island (1600 μm^2^) decreased the GTP-CDC42 levels (Figure 2D).

Aged ECs were larger than young ECs and cellular senescence detected by p16^INK4A^ staining was higher in aged ECs compared to young ECs (Figure 1C, Supplementary Figure 1C). Consistently, p16^INK4A^ intensity was higher in aged ECs cultured on large islands (2500 μm^2^) compared to that in young ECs cultured on the medium size island (1600 μm^2^), while reduction of aged EC size by culturing aged ECs on medium size islands decreased p16^INK4A^ intensity by 41% (Figure 2D). EC proliferation detected by nuclear EdU staining in aged adipose ECs cultured on the large islands (2500 μm^2^) was lower than young ECs cultured on the medium size island (1600 μm^2^), while culturing aged ECs on medium size islands restored EdU nuclear incorporation (Figure 2E). These results suggest that age-dependent increases in EC size are associated with decreased YAP1 nuclear localization, increased CDC42 activity, induction of EC senescence, and reduction of EC proliferation in aged ECs.

**Figure 3:**
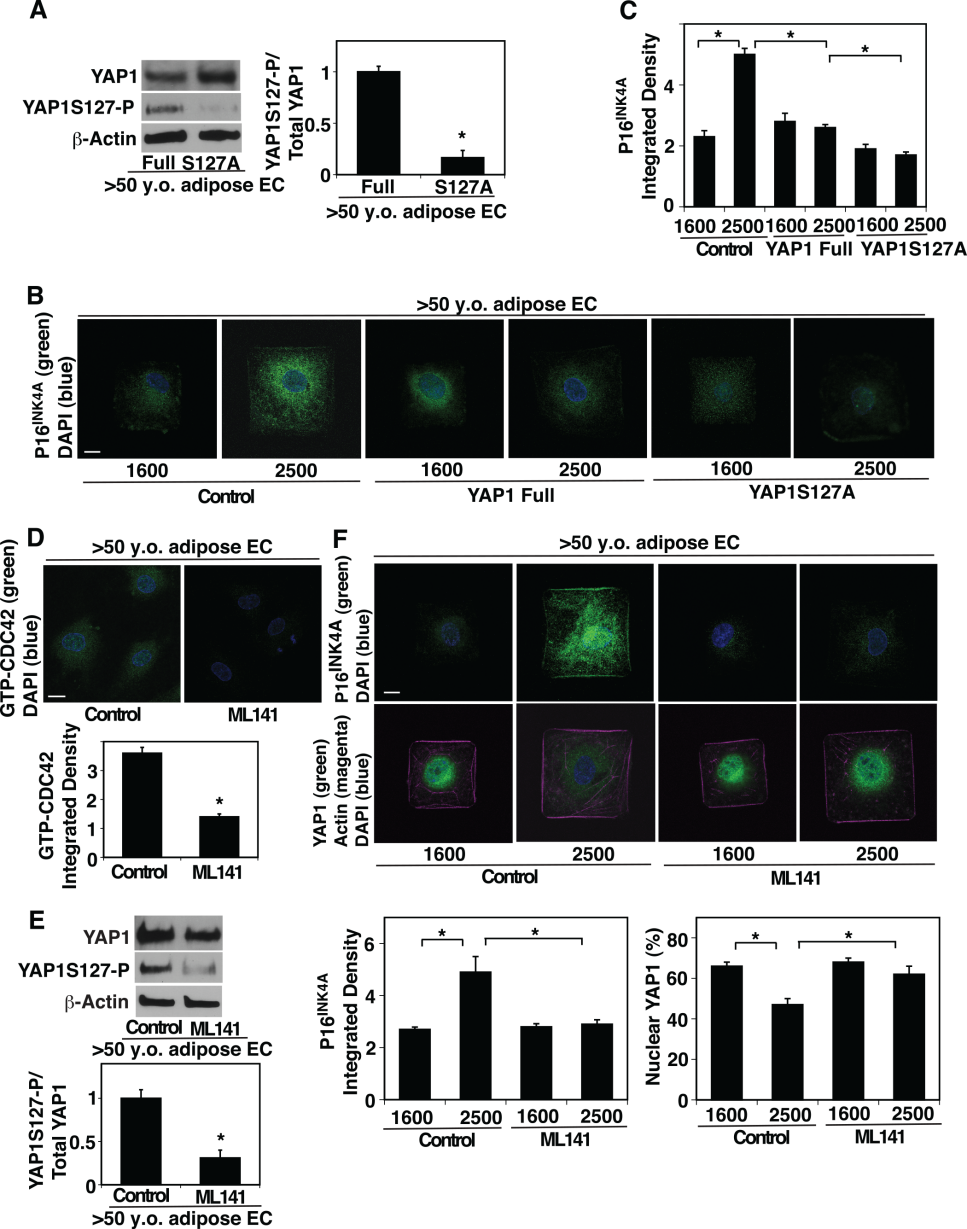
**CDC42-YAP1 mediates cell size-dependent changes in EC senescence in aged ECs.** (**A**) Representative immunoblots showing YAP1, YAP1S127 phosphorylation, and β-actin protein levels in ECs isolated from >50 y.o. human adipose tissues treated with retrovirus overexpressing full-length YAP1 or YAP1S127A (*left*). Graph showing the quantification of immunoblots (*right*, n=3, *, p<0.05). (**B**) IF micrographs showing P16^INK4A^ expression and DAPI in ECs isolated from >50 y.o. human adipose tissues treated with retrovirus overexpressing full-length YAP1 or YAP1S127A, cultured on FN-coated island of different sizes. Scale bar, 10 μm. (**C**) Graph showing quantification of P16^INK4A^ integrated density (n=7, mean ± s.e.m., *, p<0.05). (**D**) IF micrographs showing the GTP-CDC42 levels and DAPI in ECs isolated from >50 y.o. human adipose tissues treated with ML141 (500 nM). Scale bar, 10 μm. Graph showing quantification of GTP-CDC42 integrated density (n=7, mean ± s.e.m., *, p<0.05). (**E**) Representative immunoblots showing YAP1, YAP1S127 phosphorylation, and β-actin protein levels in ECs isolated from >50 y.o. human adipose tissues treated with ML-141 (*top*). Graph showing the quantification of immunoblots (*bottom*, n=3, *, p<0.05). (**F**) IF micrographs showing P16^INK4A^ expression (green) and DAPI (blue, *top*) and YAP1 localization (green), actin structure (magenta), and DAPI (blue, *bottom*) in ECs isolated from >50 y.o. human adipose tissues treated with ML141 and cultured on FN-coated island of different sizes. Scale bar, 10 μm. Graphs showing quantification of P16^INK4A^ integrated density (*bottom left*) and nuclear YAP1 (*bottom right*) (n=7, mean ± s.e.m., *, p<0.05).

To examine whether YAP1 and CDC42 mediate the effects of age-dependent increase in EC size on EC proliferation and senescence, we manipulated YAP1 activity in aged ECs and cultured them on the island of different sizes. YAP1S127A mutant construct, in which YAP1 S127 phosphorylation residue is mutated to alanine and acts as a constitutively active form of YAP1 [29, 48], inhibited YAP1S127 phosphorylation in aged human adipose ECs compared to that in full-length YAP1-treated control ECs (Figure 3A). YAP1S127A mutant construct suppressed EC senescence detected by p16^INK4A^ ICC analysis even when these aged ECs were cultured on the large island (2500 μm^2^) (Figure 3B, 3C). We also examined the effects of inhibition of YAP1 activity on cellular senescence and proliferation in young ECs. Lentiviral transduction of YAP1S94A mutant construct, which does not bind to TEAD transcription factor and acts as a dominant negative form of YAP1 [23], increased the levels of P16^INK4A^ in young ECs compared to those treated with full-length YAP1 (Supplementary Figure 2F, 2G). YAP1S94A mutant construct also decreased young EC proliferation evaluated using an EdU proliferation assay (Supplementary Figure 2H).

Inhibition of CDC42 activity by treatment with a potent selective inhibitor of CDC42, ML141 (Figure 3D) decreased YAP1S127 phosphorylation by 72% in aged ECs compared to those without ML141 treatment (Figure 3E). ML141 stimulated YAP1 nuclear localization in aged ECs cultured on the large island and inhibited EC senescence; the levels of nuclear YAP1 was 1.2-times higher and the intensity of senescence marker p16^INK4A^ was 45% lower in aged ECs treated with ML141 cultured on the large island compared to those without ML141 treatment (Figure 3F). These results suggest that YAP1 and CDC42 are responsible for age-dependent changes in EC size and control EC senescence.

### YAP1 and CDC42 mediate age-dependent decline in angiogenesis

**Figure 4:**
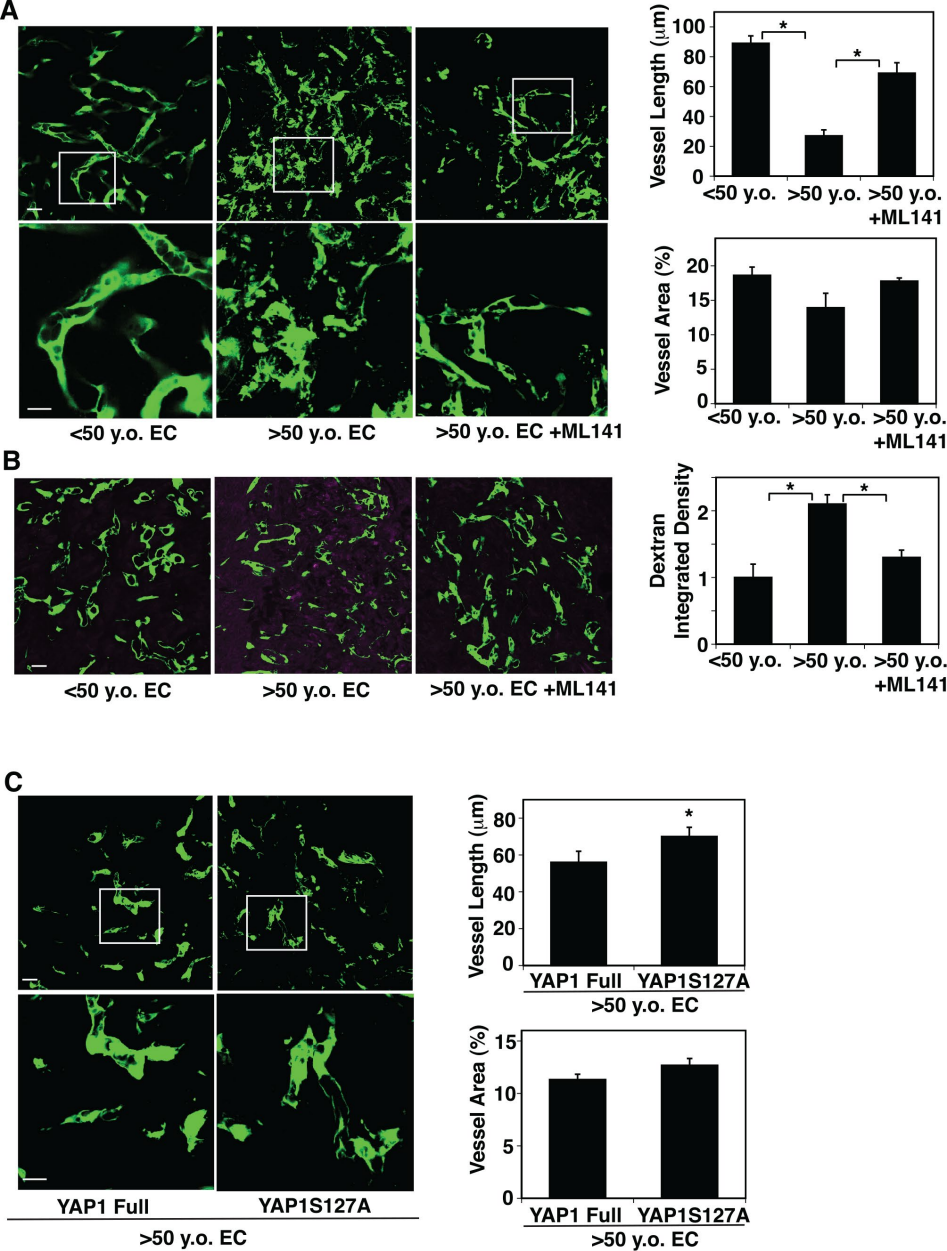
**CDC42-YAP1 signaling mediates age-dependent decline in blood vessel formation in subcutaneously implanted gel.** (**A**) IF micrographs showing vascular structures formed in the subcutaneously implanted fibrin gel supplemented with GFP-labeled ECs isolated from <50 y.o. or >50 y.o. human adipose tissues or in combination with treatment with ML141 (500 nM). Scale bar, 10 μm. Graphs showing quantification of vessel length (*top*) and vessel area (*bottom*) in the gel (n=7, mean ± s.e.m., *, p<0.05). (**B**) IF micrographs showing low MW fluorescently labeled dextran leakage (magenta) and GFP-labeled blood vessel formation (green) in the subcutaneously implanted fibrin gel supplemented with GFP-labeled ECs isolated from <50 y.o. or >50 y.o. human adipose tissues or in combination with treatment with ML141 (500 nM). Scale bar, 10 μm. Graph showing quantification of fluorescently labeled dextran leakage in the gel (n=7, mean ± s.e.m., *, p<0.05). (**C**) IF micrographs showing vascular structures formed in the subcutaneously implanted fibrin gel supplemented with GFP-labeled ECs isolated from >50 y.o. human adipose tissues in combination with treatment with retrovirus overexpressing full-length YAP1 or YAP1S127A mutant construct. Scale bar, 10 μm. Graphs showing quantification of vessel length (*top*) and vessel area (*bottom*) in the gel (n=7, mean ± s.e.m., *, p<0.05).

Angiogenesis is impaired in the aged mouse lungs [51]. YAP1 and CDC42 sense the age-dependent changes in EC size and control EC senescence (Figures. 2, 3). Therefore, we next examined whether YAP1 and CDC42 mediate age-dependent impairment of angiogenesis using a mouse gel implantation system [9, 23]. When we subcutaneously implanted fibrin gel supplemented with GFP-labeled human adipose ECs and human fibroblasts on the back of the NSG mouse [23], GFP-labeled human young adipose ECs supplemented into the gel formed a well-organized vascular lumen structures in the gel 7 days after implantation as analyzed using confocal fluorescence microscopy (Figure 4A). In contrast, GFP-labeled aged adipose ECs supplemented in the gel formed a disorganized vasculature with randomly oriented filopodia in the gel; vessel area was not significantly different in the gel supplemented with young vs. aged ECs, while vessel length was 76% shorter in aged ECs (Figure 4A). A CDC42 inhibitor, ML141, restored blood vessel structures in the gel supplemented with aged human adipose ECs; aged EC-derived blood vessels were 2.5-times longer in the gel treated with ML141 (Figure 4A). When we subcutaneously implanted gel supplemented with young vs. aged ECs and perfused with Alexa 594-labeled dextran by systemic injection, injected dextran was leaked more in the gel supplemented with aged ECs compared to that supplemented with yound ECs, while ML141 attenuated the leakage of dextran (Figure 4B), suggesting that inhibition of CDC42 restores vascular function in aged ECs in the gel.

Overexpression of YAP1S127A in aged human ECs also reversed the disorganized vascular formation in the gel compared to that supplemented with full-length YAP1-treated control ECs; although vessel area was not significantly different in the gels supplemented with aged ECs overexpressing YAP1 full and YAP1S127A, vessel length increased by 1.3-fold in aged EC-derived blood vessels treated with YAP1S127A (Figure 4C). These findings imply that lower YAP1 activity and higher CDC42 activity mediate age-dependent impairment of angiogenesis.

## DISCUSSION

In this report, we have demonstrated that aged mouse and human ECs are significantly larger than young ECs. The levels of YAP1 activity decrease, while CDC42 activity increases in aged ECs. When we culture aged human adipose ECs on single-cell sized FN-coated large islands, YAP1 is excluded from the nucleus, while reduction of the aged EC size restores YAP1 nuclear localization. Reduction of aged EC size also decreases CDC42 activity, stimulates EC proliferation and attenuates EC senescence. Inhibition of CDC42 activity decreases YAP1S127 phosphorylation and the YAP1S127A mutant construct or CDC42 inhibitor attenuates cellular senescence of aged ECs cultured on the large island. YAP1S127A or the CDC42 inhibitor also restores blood vessel structures disrupted in aged ECs in the subcutaneously implanted gel. These results suggest that age-dependent increases in EC size impair EC proliferation, induce EC senescence, and disrupt blood vessel formation through aberrant CDC42-YAP1 signaling.

This study includes multiple novelties: (1) we directly measured the size of young v.s. aged human and mouse ECs from various origins, such as small blood vessels from human adipose tissues, large blood vessels from mouse descending aorta, and pulmonary blood vessels from mouse PA ex vivo. We found that aged ECs are consistently larger compared to young ECs. Although it is reported that senescent or highly passaged cultured cells are larger [13, 14], to the best of our knowledge, this is the first report measuring the young vs. aged EC size in the ex vivo blood vessels, which may more accurately reflect the response in blood vessels in vivo. (2) Although YAP1 is known to mediate cell size-dependent signaling [18, 24, 25], the involvement of YAP1 signaling in age-dependent changes in cell size using young vs. aged ECs has not been explored. It has been reported that large cells age faster than small cells and decrease lifespan [16] and that vascular aging has significant impact on lifespan [52]. Thus, modulation of aged EC size and/or manipulation of the activity of YAP1 and CDC42 may lead to the development of promising therapeutic strategy for age-related diseases and would also be a strategy to delay the aging processes and extend lifespan.

We have demonstrated that nuclear YAP1 is lower when aged EC was cultured on a large island (mimics aged cell size), while YAP1 localizes in the nucleus when the aged cell size is reduced by culturing on a medium size island (mimics young cell size). However, when cell size is further reduced by culturing on a smaller size island, YAP1 activity decreases. Consistently, P16^INK4A^ levels are high when aged EC was cultured on a large island. P16^INK4A^ levels decrease when the aged cell size is reduced by culturing on a medium size island. However, when cell size is further reduced, P16^INK4A^ levels increase again. Thus, nuclear YAP1 correlates with P16^INK4A^ levels and appropriate medium cell size is necessary for YAP1 nuclear translocation and lowering P16^INK4A^ levels in aged ECs. Regarding GTP-CDC42, consistent with nuclear YAP1, the levels of GTP-CDC42 are higher when aged EC was cultured on a large island. The GTP-CDC42 levels decrease when the aged cell size is reduced by culturing on a medium size island. However, inconsistent with nuclear YAP1, even when cell size is further reduced by culturing on a smaller size island, the GTP-CDC42 levels did not increase. This may be because other Rho small GTPases or actin cytoskeleton-related proteins are involved in the mechanism in aged cells in the range of cell size. Alternatively, reduction of YAP1 activity in aged ECs on the small island may feedback to inhibit CDC42 activity.

Aged tissues are exposed to aberrant ECM stiffness [12] and blood flow [5], which changes cell size [53] and contributes to various age-dependent cardiovascular diseases including hypertension and atherosclerosis [54, 55]. We have reported that changes in ECM stiffness alter EC shape and size, and consequently modulate angiogenic gene expression, EC proliferation, behaviors, and function [9–11]. Thus, age-dependent increases in EC size induced by aberrant micromechanical environment may disturb angiogenesis and contribute to various diseases in aged adults, and investigation of the signaling mechanism by which EC size directly controls EC behaviors using the microcontact printing system would further our understanding of the mechanism of aging. In addition to EC size, cell geometry, adhesion area, and ECM components also change cytoskeletal structure and may affect YAP1 and CDC42 activity and related signaling (*e.g.,* Rho) [8, 17, 18, 29, 50, 56–58]. Culturing ECs on microcontact printed islands of different shapes (circle, square, triangle, rectangle) [24, 25, 56, 57], different adhesion area [24, 57, 58], or coated with different ECMs (*e.g.,* collagen) will elucidate the mechanism.

We have demonstrated that YAP1 and CDC42 mediate aged EC size-dependent inhibition of EC proliferation and angiogenesis. Another Hippo signaling molecule, transcriptional co-activator with PDZ-binding motif (TAZ), which has similar molecular architectures and controls angiogenesis [20], has distinct biological activities from YAP1 [59]. YAP1/TAZ also control cell size and mechanics [25], and there may be a feedback mechanism. In addition, cell size controls EC proliferation through other mechanosensitive genes. For example, mechanosensitive transcription factors (e.g., TFII-I, GATA2, TWIST1) control angiogenesis and EC integrity, and contribute to angiogenesis-related diseases (*e.g.,* pulmonary fibrosis, pulmonary hypertension) [9, 60, 61]. Wnt co-receptor LRP5, which controls YAP1/TAZ activity [62], mediates ECM stiffness-induced Tie2 expression in ECs, and modulates lung development and age-dependent inhibition of post-PNX lung growth by changing angiogenesis [51, 63]. The expression of TWIST1 is regulated by YAP1 [64] and controls cellular senescence [65]. YAP1 also controls angiogenesis through PGC1α [23], which regulates mitochondrial biogenesis and metabolism [66] and contributes to aging processes [67]. It has been reported that increasing organismal size correlates with lower oxygen consumption in mitochondria and cellular functionality [15], and therefore age-dependent increases in cell size may suppress cell proliferation in aged adults by impairment of cellular metabolism as well. Further characterization of the YAP1-related mechanosensitive signaling pathways will elucidate the mechanism.

Our results suggest that increases in aged EC size stimulate CDC42 activity, which results in the suppression of YAP1 activity and induces EC senescence. This seems to contradict others’ reports showing that YAP1 is in the nucleus and active in spreading cells (*i.e.,* cells are larger) [24, 48]. The response of YAP1 activity to cell size would be different between young cells and aged cells. Although we have demonstrated that inhibition of CDC42 activity stimulates YAP1 activity in aged ECs (Figure 3), it is reported that inhibition of CDC42 suppresses YAP1 activity in alveolar type II cells during lung regeneration after PNX in young adult mice [43]. The response of YAP1 to CDC42 may be different among cell types and ages of the cells. CDC42 controls YAP1 activity but YAP1 also induces CDC42 activity and controls developmental angiogenesis and vascular integrity [20, 22]. A complex feedback mechanism to control YAP1 and CDC42 activity may be involved in the mechanism. Other CDC42-related signaling molecules (e.g., Rho, Rac, integrins) that control actin cytoskeleton in a distinct way also interact with each other and regulate EC proliferation in a context dependent manner [8, 50, 56–58]. For example, increases in Rho activity stimulate YAP1 activity and inhibit stem cell apoptosis [68]. Integrin signaling, which modulates cell size and shape, mediates mechanical force-dependent YAP1/TAZ activity in various tissues [69–71]. Spatiotemporal control of YAP1/CDC42 activity and maintenance of appropriate cell size will be required to maintain young blood vessel structures in the organs.

It remains unclear the mechanism by which aging increases EC size. Multiple factors such as changes in ECM components and mechanics, oxygen stress, nutrients, and compensatory response to damaged cells (cell competition) [72] would be involved in the mechanism. Recently, it has been reported that fibroblast membranes extend to fill the empty space of lost neighboring fibroblasts rather than proliferation or migration during homeostasis and aging [73]. In addition to ECs, epithelial cells and other cells (e.g., smooth muscle cells, fibroblasts, immune cells) secrete angiogenic and other chemical factors [74], which may indirectly control EC size and shape in aged tissues. Further analysis of the effects of aging on the size of ECs and other cell types will be necessary to elucidate the mechanism by which aging impairs angiogenesis and epithelial morphogenesis.

We have investigated the effects of aged cell size on EC proliferation and senescence using ECs isolated from human adipose tissues with a variety of conditions that can affect EC size and angiogenic activity. We excluded the samples from cancer patients but other diseases were included in the cohort. The average BMI of the study population is higher than 30 kg/m^2^. However, when we analyzed the EC size in young vs. aged lean group (BMI, <30 kg/m^2^) and obese group (BMI, >30 kg/m^2^), aged ECs are significantly larger compared to young ECs in both lean and obese groups (not shown), suggesting that age-dependent changes in EC size may not be dependent on the level of obesity. The heterogeneity of the samples due to cardiovascular condition (e.g., hypertension, hyperlipidemia, diabetes mellitus), sex, and collected regions of the body (visceral, subcutaneous) may impact the EC size and subsequent signaling pathways. Further investigation in another cohort with a larger sample size will be necessary to elucidate the effects of aged EC size on angiogenesis.

In summary, we have demonstrated that age-dependent increases in EC size induce EC senescence and suppress angiogenesis through CDC42-YAP1 signaling. Modulation of EC size or YAP1 or CDC42 activity would potentially lead to the development of new therapeutic strategies for aging-related diseases.

## MATERIALS AND METHODS

### Materials

The following reagents were used: Anti-paxillin and -VE-cadherin antibodies (Transduction Laboratories, Lexington, KY); anti-BrdU, –p16^INK4A^, and -phospho-YAP1 (Ser 127) antibodies (Abcam, Cambridge, MA); anti-β-actin monoclonal antibody (Sigma, St. Louis, MO); anti-YAP1 antibody (Santa Cruze Biotechnology, Dallas, TX); anti-GTP-CDC42 antibody (NewEast Biosciences, King of Prussia, PA); anti-YAP1 and -CDC42 antibodies (Cell Signaling Technology, Danvers, MA); ML141 (Sigma).

### Human adipose tissue acquisition

Fresh human subcutaneous adipose tissues (n= 55 people) were obtained as discarded surgical specimens from patients undergoing abdominal surgeries. After surgical removal, samples were placed in ice-cold HEPES buffer and immediately transferred to the laboratory for isolated vessel studies. De-identified patient demographic data were collected using the Generic Clinical Research Database (GCRD) at the Medical College of Wisconsin. All protocols were approved by the Institutional Review Board of the Medical College of Wisconsin and Froedtert Hospital. Sample demographic information is summarized in Table 1. The patients with any types of cancer were excluded.

### Mouse lung EC isolation

Mouse pulmonary artery and descending aorta were dissected from C57BL6 mice of different ages (2M and 24M old). Mouse lung ECs were isolated from C57BL6 mice of different ages (2M and 24M old) using anti-CD31 conjugated magnetic beads and sorted by FACS (CD31^+^, VE-Cadherin^+^, CD45^-^) as previously reported [51]. Isolated ECs were validated as ECs by FACS. Isolated mouse ECs were cultured in EBM2 medium containing 5% FBS and growth factors (VEGF, bFGF and PDGF) [51] and were used between passages 1-2.

### Plasmid construction and gene knockdown

The retroviral full-length pQCXIH-myc-YAP1 (human) and pQCXIH-flag-YAP1-S127A (human) were gifts from Kunliang Guan (Addgene plasmid # 33091 and # 33092) [75]. pLX304-YAP1 (S94A) (human) construct was a gift from William Hahn (Addgene plasmid # 59145) [76]. As a control, plasmid with vector only or full-length YAP1 construct was used. Generation of retroviral vectors was accomplished as reported [9]. Viral supernatants were collected starting 48 h after transfection, for four consecutive times every 12 h, pooled, and filtered through a 0.45 μm filter. Viral supernatants were then concentrated 100-fold by ultracentrifugation in a Beckman centrifuge for 1.5 h at 16,500 rpm. Human adipose ECs were incubated with viral stocks in the presence of 5 μg/ml polybrene (Sigma) and 90–100% infection was achieved 3 days later [9, 23]. The ratio of the levels of exogenous YAP1 to endogenous YAP1 in the aged ECs retrovirally transduced YAP1 and YAP1S127A were 3.2 and 5.6, respectively (not shown), indicating that retroviral transduction of these proteins overcomes the endogenous effects.

### Cell biological methods

Blood vessels isolated from mouse pulmonary artery, mouse descending aorta, or human adipose tissues were stained with silver nitrate as previously reported [44, 45]. Under the dissection microscope, we dissected blood vessels with a length of circumference of 300 μm (a diameter of approximately 50 μm) from the adipose tissue. The blood vessels were cannulated with 25G metal blunt needle and consecutively perfused with 2% PFA, 5% glucose, 0.2% AGNO3, 5% glucose and 2% PFA. After perfusion, the blood vessels were cut open and mounted on the slide. Under light microscope, endothelial cell-cell junctions were visualized and cell size was measured on at least five images using ImageJ software (NIH). For cultured ECs, we immunostained ECs with VE-cadherin and imaged cell-cell junctions using a confocal Leica SP5 microscope. Cell size was measured on at least five images using ImageJ software.

The ECs were isolated as described before [77]. Briefly, after removing the fibrotic and cauterized regions, about 5 g of subcutaneous adipose tissue was minced using small scissors and transferred into 15 ml tubes containing 5 ml of 1 mg/ml Collagenase A (Roche, Basel Switzerland). The sample was digested by intermittent pipetting for 30 min at 37°C and filtered through 40 μm nylon mesh. The cells were washed with PBS and treated with RBC lysis buffer (Sigma). After washing with PBS, the cells were mixed with CD31-conjugated magnetic beads (Dynabeads®, Invitrogen) and the ECs were isolated according to the manufacture’s protocol and sorted by FACS (CD31^+^, VE-Cadherin^+^, CD45^-^) as previously reported [51]. Isolated ECs were validated as ECs by FACS. Isolated human adipose ECs were cultured on attachment factor (Cell Systems, Kirkland, WA)-coated dish with endothelial basal medium (EBM2) containing 5% FBS and growth factors (VEGF, bFGF and PDGF). Isolated ECs were used between passages 1-3.

EC proliferation was analyzed by a BrdU incorporation assay or a Click-iT® Plus EdU Proliferation Assay. Mouse lung and human adipose ECs were plated in EBM2 with 2% serum, pulsed with 5 μM BrdU for 16 h, immunostained and imaged using a confocal Leica SP5 microscope. Cellular senescence was characterized using a SA-β galactosidase assay kit (cell signaling) or anti-P16^INK4A^ staining. The specificity of P16^INK4A^ staining was confirmed by 2^nd^ Ab alone staining (Supplementary Figure 1D). The microscopic images were analyzed on at least five images using imageJ software (NIH) and the same contrast and brightness were used to compare the images.

### Molecular biological and biochemical methods

RNA was isolated using an RNeasy mini kit (Qiagen, Valencia, CA, USA). Quantitative reverse transcription (qRT)-PCR was performed with the iScript reverse transcription and iTaq SYBR Green qPCR kit (BioRad, Hercules, CA) using the BioRad real time PCR system (BioRad). Cyclophilin and β2 microglobulin (B2M) controlled for overall cDNA content. The primers used were human *P16^INK4A^*; forward 5′-GATCCAGGTGGGT AGAAGGTC-3′, reverse 5′-CCCCTGCAAACTTCG TCCT-3′; mouse *P16^Ink4a^*, forward 5’-CGCAGGTTCT TGGTCACTGT-3′, reverse 5′- TGTTCACGAAAGC CAGAGCG-3′. The primers used for human and mouse *YAP1*, mouse cyclophilin and human B2M were previously described [9, 23]. CDC42 activity was measured using the CDC42 pull-down activity assay kit (Cytoskeleton, Denver, CO).

### Microcontact printing system

Stamps were created using soft lithography as described previously [10, 49, 50]. Polydimethylsiloxane (PDMS) stamps were made by casting the polymer against master molds made by standard photolithography using the negative photoresist SU-8 (MicroChem). Substrates for stamping were fabricated by spin-coating a thin layer of PDMS (Sylgard-184, Dow Corning) onto glass coverslips. To coat a coverslip, a drop of PDMS (200 μl for a 25 mm×25 mm coverslip, Corning) was applied to the center of the coverslip and spun at 4000 rpm for 4 minutes on a spin-coater (Specialty Coating Systems G3-8, Cookson Electronics) and cured at 60°C for one hour. Prior to stamping, PDMS stamps were cleaned in 70% ethanol in a sonicating water bath for 30 minutes, rinsed with water, and dried using filtered compressed air or nitrogen gas. The surface of the clean stamps containing the raised micropatterned features were incubated with 50 μg/ml FN in aqueous solution for one hour, and dried thoroughly with filtered nitrogen gas or compressed air. Directly before use, the PDMS-coated coverslips were activated by oxygen plasma in a UVO cleaner (Jelight) for 8 minutes, during which time inked PDMS stamps were dried. The stamps were then pressed gently against the plasma-treated PDMS surface to ensure complete contact of stamp with substrate. Unstamped areas were blocked by incubation in 1% Pluronic-127 for 1 hour at room temperature or overnight at 4°C. Before plating cells, substrates were washed three times with PBS to remove residual Pluronic-127. 1X10^4^ ECs in 1.5 ml culture medium were plated on the coverslips (22 mm X 22 mm), which allows each cell to fit on each single FN-island on the coverslip. The cells were cultured for 16 hours.

### Mouse subcutaneous fibrin gel implantation

The *in vivo* animal study was carried out in strict accordance with the recommendations in the Guide for the Care and Use of Laboratory Animals of the National Institutes of Health. The protocol was reviewed and approved by the Animal Care and Use Committee of Medical College of Wisconsin. NOD scid gamma (NSG) mice (8 week old; Jackson Laboratory) and C57BL6 mice (Jackson Laboratory and NIA/NIH rodent colonies) were used for the study. Fibrin gel was fabricated as described [51, 60, 61]. Briefly, we added 20 μl of thrombin (2.5 U/ml) to 20 μl of fibrinogen solution (12.5 mg/ml) and supplemented gel with GFP-labeled human adipose ECs (1X10^6^ cells), in which gene expression was manipulated, and human dermal fibroblasts (3X10^5^ cells, ATCC). The drops of the gel were incubated at 37 °C for 30 min until they solidified [60, 61]. For treatment with ML141, we mixed the gel with ML141 (final concentration; 500 nM). We then implanted the gel subcutaneously on the back of NSG mice for 7 days as previously described [9, 23]. Vascular network formation of GFP-labeled human adipose ECs was evaluated by measuring the area and length of GFP-labeled blood vessels from five different areas of the gel [9, 60, 61]. The vascular permeability was measured using low MW fluorescently labeled dextran (MW 4000, Sigma) leakage [11]. Fluorescent images were taken on a Leica TCS SP5 confocal laser scanning microscope and morphometric analysis was performed using ImageJ and Angiotool softwares [9, 60, 61].

### Statistical analysis

All phenotypic analysis was performed by masked observers unaware of the identity of experimental groups. Error bars (SEM) and *p* values were determined from the results of three or more independent experiments. The F test (for two samples) or the Levene test (for more than two samples) was performed to confirm that the variances are homogeneous. Student’s t-test was used for statistical significance for two groups. For more than two groups, one-way ANOVA with a post-hoc analysis using the Bonferroni test was conducted.

## Supplementary Material

Supplementary Figures
