# Supplementary material for: Effects of age-dependent changes in cell size on endothelial cell proliferation and senescence through YAP1
Source: Aging (Albany NY). 2019 Sep 5;11(17):7051–69. doi: 10.18632/aging.102236 (PMC6756888; doi:10.18632/aging.102236)
Supplement: Supplementary Figures [file aging-11-102236-s001.pdf]

SUPPLEMENTARY FIGURES

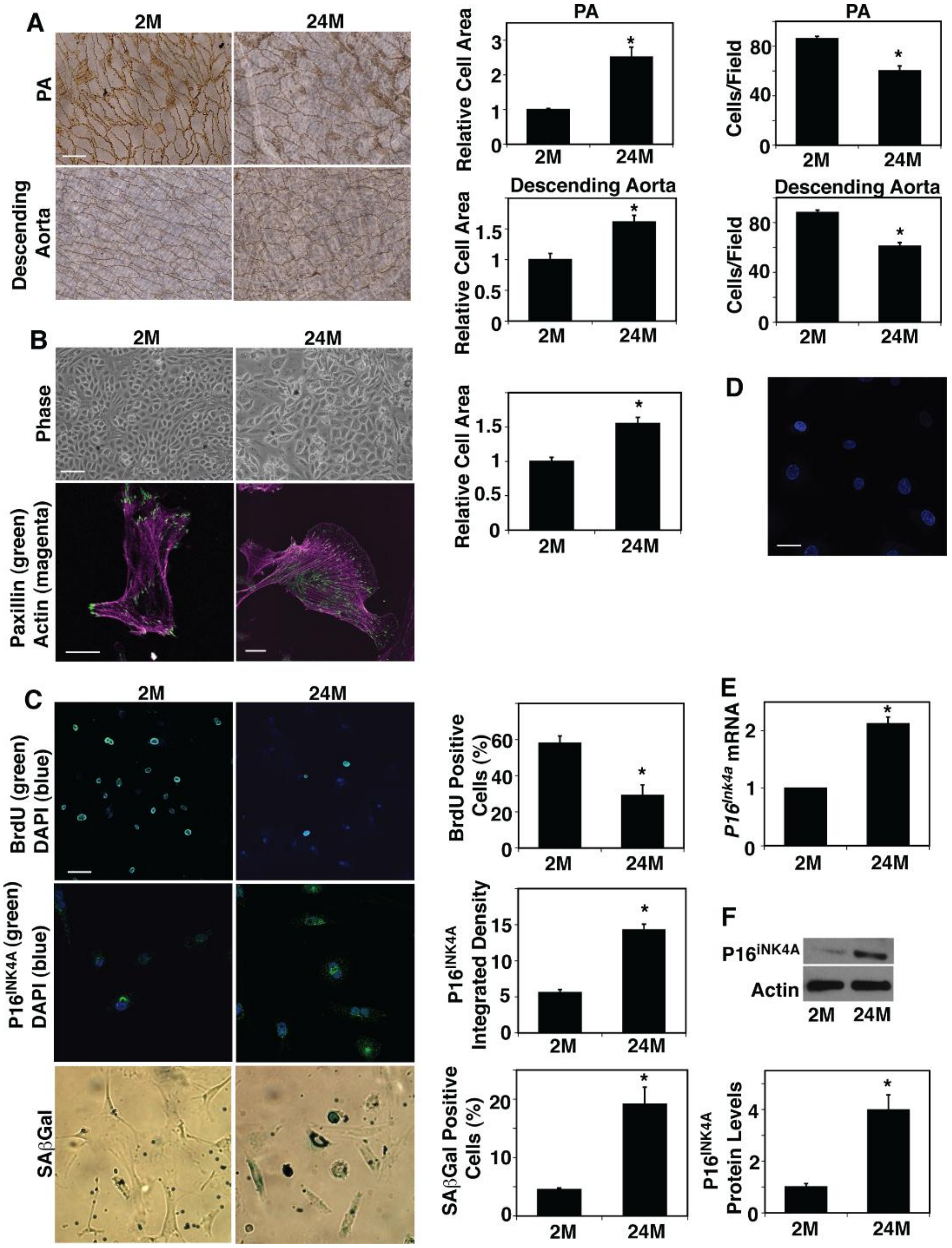

Supplementary Figure 1. Age-dependent changes in mouse EC size, proliferation and senescence. (**A**) Silver nitrate-stained 2M and 24M old mouse PA (*top*) and descending aorta (*bottom*). Scale bar, 20 µm. Graphs showing quantification of cell area and density in PA (*top*) and descending aorta (*bottom*) dissecting from 2M and 24M old mice (n=7, mean ± s.e.m., *, p<0.05). (**B**) Phase contrast images of cultured ECs isolated from 2M vs. 24M old mouse lungs (*top*, scale bar, 100 µm). IF micrographs showing paxillin-positive focal adhesions (green) and actin stress fiber formation (magenta, *bottom*). Scale bar, 20 µm. Graph showing quantification of cell area of ECs isolated from 2M and 24M old mouse lungs (n=7, mean ± s.e.m., *, p<0.05). (**C**) IF micrographs showing BrdU^{+} (*top*), P16^{INK4A+} (*middle*), and SA βGal-stained (*bottom*) ECs isolated from 2M vs. 24M old mouse lungs. Graphs showing quantification of BrdU^{+} (*top*), P16^{INK4A+} (*middle*), and SAβGal-stained (*bottom*) ECs isolated from 2M vs. 24M old mouse lungs (n=7, mean ± s.e.m., *, p<0.05). Scale bar, 20 µm. (**D**) IF micrograph showing the 2nd Ab alone (green) and DAPI (blue) staining. Scale bar, 20 µm. (**E**) Graph showing *P16^{Ink4a}* mRNA levels in ECs isolated from 2M vs. 24M old mouse lungs (n=5, mean ± s.e.m., *, p<0.05). (**F**) Representative immunoblots showing P16^{INK4A} and β-actin protein levels in ECs isolated from 2M vs. 24M old mouse lungs (*top*). Graph showing the quantification of immunoblots (*bottom*, n=3, mean ± s.e.m., *, p<0.05).

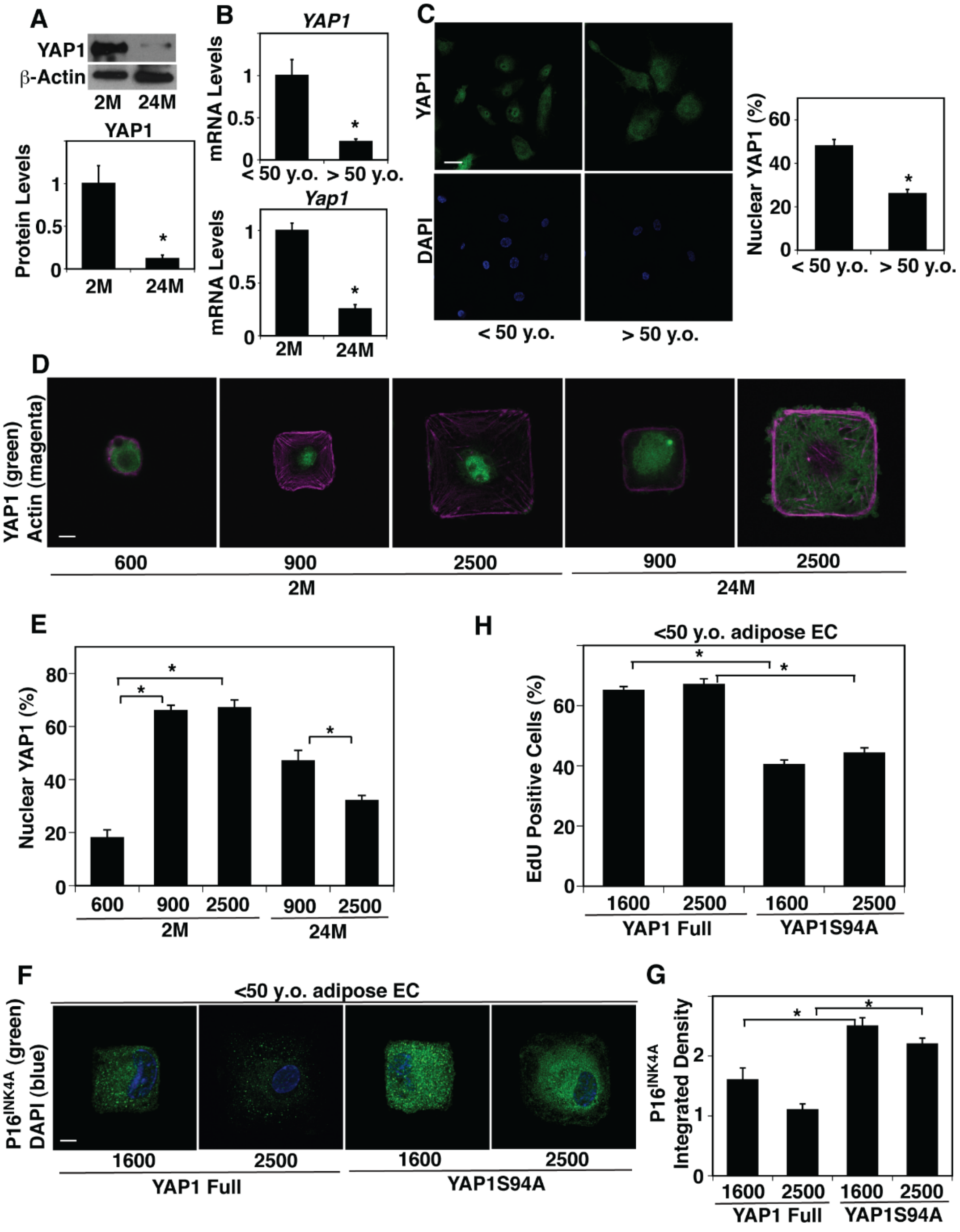

Supplementary Figure 2. Cell size-dependent changes in YAP1 activity in mouse lung ECs. (**A**) Representative immunoblots showing YAP1 and β-actin protein levels in ECs isolated from 2M vs. 24M old mouse lungs (*top*). Graph showing the quantification of immunoblots (*bottom*, n=3, *, p<0.05). (**B**) Graphs showing *YAP1* mRNA levels in ECs isolated from <50 y.o. vs. >50 y.o. human adipose tissues (*top*) and 2M vs. 24M old mouse lungs (*bottom*, n=5, mean ± s.e.m., *, p<0.05). (**C**) IF micrographs showing YAP1 localization (*top*) and DAPI (*bottom*) in ECs isolated from <50 y.o. and >50 y.o. human adipose tissues. Scale bar, 20 µm. Graph showing quantification of nuclear YAP1 in ECs isolated from <50 y.o. and >50 y.o. human adipose tissues (n=7, mean ± s.e.m., *, p<0.05). (**D**) IF micrographs showing YAP1 localization (green) and actin structure (magenta) in ECs isolated from 2M vs. 24M old mouse lungs and cultured on FN-coated island of different sizes. Scale bar, 10 µm. (**E**) Graph showing quantification of nuclear YAP1 (n=7, mean ± s.e.m., *, p<0.05). (**F**) IF micrographs showing $P16^{INK4A}$ expression (green) and DAPI (blue) in ECs isolated from <50 y.o. human adipose tissues overexpressing full-length YAP1 or YAP1S94A, cultured on FN-coated island of different sizes. Scale bar, 10 µm. (**G**) Graph showing quantification of $P16^{INK4A}$ integrated density in ECs isolated from <50 y.o. human adipose tissues overexpressing full-length YAP1 or YAP1S94A, cultured on FN-coated island of different sizes (n=7, mean ± s.e.m., *, p<0.05). (**H**) Graph showing quantification of EdU-positive ECs isolated from <50 y.o. human adipose tissues overexpressing full-length YAP1 or YAP1S94A, cultured on FN-coated island of different sizes (n=7, mean ± s.e.m., *, p<0.05).
